# Genomic prediction enables rapid selection of high‐performing genets in an intermediate wheatgrass breeding program

**DOI:** 10.1002/tpg2.20080

**Published:** 2021-03-03

**Authors:** Jared Crain, Lee DeHaan, Jesse Poland

**Affiliations:** ^1^ Dep. Plant Pathology Kansas State University 4024 Throckmorton Plant Sciences Center Manhattan KS USA 66506; ^2^ The Land Institute 2440 E. Water Well Rd Salina KS USA 67401; ^3^ Wheat Genetics Resource Center, Dep. Plant Pathology Kansas State University 4024 Throckmorton Plant Sciences Center Manhattan KS USA 66506

## Abstract

In an era of constrained and depleted natural resources, perennial grains could provide sustainable food production along with beneficial ecosystem services like reduced erosion and increased atmospheric carbon capture. Intermediate wheatgrass (IWG) [*Thinopyrum intermedium* (Host) Barkworth & D. R. Dewey subsp. *intermedium*] has been undergoing continuous breeding for domestication to develop a perennial grain crop since the 1980s. As a perennial, IWG has required 2–5 yr per selection generation, but starting in 2017, genomic selection (GS) was initiated in the breeding program at The Land Institute, Salina, KS (TLI), enabling one complete cycle per year. For each cycle, ∼4,000 seedlings were profiled using genotyping‐by‐sequencing (GBS) and genomic estimated breeding values (GEBVs) were calculated. Selection based on GEBVs identified ∼100 individuals to advance as parents each generation, while validation populations of 1,000–1,200 genets for GS model training were also selected using the genomic relationship matrix to represent genetic diversity in each cycle. The selected parents were randomly intermated in a greenhouse crossing block to form the subsequent cycle, while the validation populations were transplanted to irrigated and nonirrigated field sites for phenotypic evaluations in the following years. For priority breeding traits of seed mass, free threshing, and nonshattering, correlations between predicted values and observed data were >.5. The realized selection differential ranged from 11–23% for selected traits, and the expected genetic gains for these traits, including spike yield, ranged from 6 to 14% per year. Genomic selection is a powerful tool to speed the domestication and development of IWG and other perennial crops with extended breeding timelines.

## Core Ideas

- Three cycles of genomic selection were completed in intermediate wheatgrass.
- Realized selection differentials ranged from 11 to 23% for priority traits.
- Expected annual gains were 6–14% for free threshing, shattering, seed mass, and yield.
- Correlation of GEBVs to observed data for 37 of 46 traits was >.3.
- Genomic selection provides a tractable method to improve perennial traits.

**Abbreviations**
BLUP: best linear unbiased predictor
GBS: genotyping‐by‐sequencing
GEBV: genomic estimated breeding value
GS: genomic selection
IWG: intermediate wheatgrass
SNP: single nucleotide polymorphism
TLI: The Land Institute.

## 1 INTRODUCTION

Agriculture faces tremendous challenges in the coming decades. World population is expected to exceed 9 billion people by 2050, (Godfray et al., 2012) demanding nearly doubling food production from 2005 levels (Tilman et al., 2011). Agriculture will also be expected to provide this increased production in a more sustainable manner by requiring less land, reducing soil erosion, and increasing carbon capture (Godfray et al., 2012). Coupled with less‐favorable climates, these immense pressures will require extraordinary efforts to develop sustainable and resilient cropping systems to meet future demands.

One potential avenue to address these challenges is the development of perennial grain crops. Perennial crops could provide ecosystem benefits of reduced soil erosion by limiting the annual disruption of the soil (Crews et al., 2018), reduced nitrate leaching (Culman et al., 2013; Jungers et al., 2019), and increased carbon capture (Sprunger et al., 2018). Currently, there are no widely grown perennial grain crops, but several perennial ecotypes of annual species are being evaluated for wheat (*Triticum aestivum* L.), sorghum [*Sorghum bicolor* (L.) Moench], rice (*Oryza sativa* L.), and sunflower (*Helianthus annuus* L.) (Cox, Van Tassel, Cox & Dehann, 2010). One of the most promising candidates for a perennial grain crop is intermediate wheatgrass [*Thinopyrum intermedium* (Host) Barkworth & D. R. Dewey subsp. *intermedium*] (IWG), which is marketed under the trade name Kernza (DeHaan & Ismail, 2017).

Intermediate wheatgrass was selected for domestication from an evaluation of over 100 perennial grasses in the 1980s by work at the Rodale Institute, Kutztown, PA (Wagoner, 1990). Since that time, five breeding programs have been established with material originally developed by the Rodale Institute and USDA's Big Flats Plant Material Center, Corning, NY (Zhang et al., 2016). The Land Institute (TLI) at Salina, KS, has been a leader in domesticating IWG, taking over breeding from Rodale in 2001 and completing six cycles of selection by 2017. This breeding work has mainly focused on improving key domestication and agronomic traits such as grain yield, free‐threshing seed, and reducing shattering (DeHaan et al., 2018). These results have been promising, with estimated accumulated gains reaching 143, 181, and 60% over five cycles of selection for seed yield, increased free‐threshing, and increased seed size, respectively (DeHaan et al., 2018). Even with these impressive gains, it is predicted that at least 20 yr of sustained genetic gain will be required to achieve grain yields similar to wheat and 100 yr to achieve the same seed mass as wheat (DeHaan et al., 2014).

Genomic selection (GS) is a breeding method that uses dense molecular markers so that each quantitative trait loci (QTL) is in linkage disequilibrium with a marker, allowing the total genetic value of individuals to be predicted only from genotypic information (Meuwissen et al., 2001). As IWG is a perennial crop, the phenotypic breeding cycle is often multiple years (DeHaan et al., 2018), allowing GS to potentially reduce breeding cycle time within this crop (Heffner et al., 2010; Wong & Bernardo, 2008). Zhang et al. (2016) published the first account of GS in IWG and showed that GS could have high predictive ability and increase the rate of domestication in IWG. Work at TLI showed that GS could result in up to a 2.6‐fold increase in genetic gain (Crain et al., 2021) for spike yield compared with phenotypic selection. Using 8 yr of data spanning five cycles of selection and two breeding programs (TLI and University of Minnesota), Crain et al. (2020a) showed that key domestication traits, such as free‐threshing and reduced shattering, had high predictive ability regardless of the environment used to train the GS model. Other work has shown that incorporating genome‐wide association studies and genotype‐by‐environment interactions into GS models can increase the accuracy of GS prediction in IWG (Bajgain et al., 2019, 2020).

While this growing body of research has shown the power of GS within IWG, all of these studies have looked at prediction accuracy with cross‐fold validation within datasets but have not made or evaluated forward predictions to the next cycle in the breeding program. In order to reduce the domestication timeline and time to a perennial grain crop, TLI adopted GS within the breeding program beginning in 2017. Using the breeding strategy presented in Crain et al. (2021), the breeding cycle has been reduced to 1 yr, with breeding Cycles 7, 8, and 9 being completed in 2017, 2018, and 2019, respectively. Thus three cycles of selection have been completed in the time that would be required to complete one cycle of phenotypic selection with 2 yr of phenotypic data evaluation (DeHaan et al., 2018).

Here we evaluate the effectiveness of the TLI GS program. Specifically, our objectives are to (a) determine the forward prediction accuracy between GS predictions and observed values, (b) examine the challenges of completing one cycle of selection per year, and (c) evaluate the trait means of GS selected plants vs. unselected plants to estimate the rate of genetic gain.

**Core Ideas**

- Three cycles of genomic selection were completed in intermediate wheatgrass.
- Realized selection differentials ranged from 11 to 23% for priority traits.
- Expected annual gains were 6–14% for free threshing, shattering, seed mass, and yield.
- Correlation of GEBVs to observed data for 37 of 46 traits was >.3.
- Genomic selection provides a tractable method to improve perennial traits.

## 2 MATERIALS AND METHODS

### 2.1 Plant material and phenotypic evaluation

Breeding cycles used in this study include material from TLI‐Cycles 6 through 9, with each cycle described below. To maintain consistency with Zhang et al. (2016), we will refer to genetically unique individuals as genets, with genotype referring to the genetic makeup of the genet. Typically, genets are initially single plants, but for perennial species they can be cloned to create multiple individual plants with identical genetic makeup, which are then termed ramets.

### 2.2 TLI‐Cycle 6

The formation of TLI‐Cycle 6 has been described in detail in DeHaan et al. (2018). Briefly this cycle of selection consisted of 20,360 genets representing 365 unique full‐sib families from 109 unique parents. Genets were started in the greenhouse and transplanted to the field in the fall of 2015. Genets were unreplicated, and no common checks were used based on the difficulty of cloning sufficient number of ramets of check genets. The genets were arranged in a grid with 91 cm between rows and 61 cm between columns allowing for a spatial correction based on location within the grid. Phenotypic evaluation for a wide range of traits was completed during the summers of 2016 and 2017, with emphasis on priority traits of free‐threshing seed, reduced shattering, spike yield, and seed mass. For all cycles of data, we analyzed multiple years of the same trait type, that is, shattering observed in 2016 and 2017 separately.

After 2 yr of phenotypic evaluation, 89 TLI‐Cycle 6 plants were selected as parents to begin the TLI‐Cycle 7 generation. These parents were selected by ordering the best linear unbiased predictors (BLUPs; described below) of the traits for each genet, with emphasis on priority traits, and choosing individuals that possessed the best values for most of the traits under selection (DeHaan et al., 2018). These 89 genets (individual plants) were dug from the field and cloned four to eight times into individual pots. To prevent relative maturity from affecting random intermating, the ramets were separated into two groups by genet, with the first and second group of genets entering the greenhouse 2 wk apart. The plants were randomly placed on capillary watering mats on the floor and rearranged every 3–5 d during anthesis to favor random intermating. Oscillating fans were also used to aid pollen dispersion within the greenhouse. An average temperature of 20.6 °C was maintained in the greenhouse, with a range between 16.7 and 25.0 °C. Day length was maintained at 16 h with supplemental lighting provided with 1000‐W metal halide lamps when ambient light was below 240 umol m^2^ s^−1^ photosyntetically active radiation.

### 2.3 TLI‐Cycle 7

Cycle 7 seed was harvested and ∼50 seeds from each maternal half‐sib family were started in Q Plugs (International Horticultural Technologies) sized 30 mm in diameter by 55 mm deep in late July 2017. Tissue collection for genotyping (genetic profiling) began in early September 2017, with ∼45 genets per each mother being selected based on visual selection to remove seedlings with low vigor. In total, 4,170 genets were sampled for DNA extraction and genomic selection (GS).

Using genomic estimated breeding values (GEBVs, output from a GS model; described below) derived from a training model using only TLI‐Cycle 6 phenotypic data, we selected genets with emphasis on free threshing, shattering, spike yield, and seed mass to identify 118 genets to be the parents for TLI‐Cycle 8. Selection of these parents was similar to TLI‐Cycle 7 parents with manual selection index. These parent plants were transplanted to 3.8‐L pots, allowed to tiller, and then divided into four ramets each that were established in separate 3.8‐L pots. To induce flowering, selected plants were placed in a vernalization chamber set at 4 °C with 10 h days. Two ramets of each genet were vernalized for 7 wk, and the second two for 9 wk so that early and late‐flowering genets would have opportunity to intermate. Random intermating in the greenhouse was accomplished in the same manner as described in TLI‐Cycle 7.

After the parent plants were identified, we used the genomic relationship matrix (**K**; genomic prediction) to identify genets to place in the TLI‐Cycle 7 validation population. In order for the validation population to represent the selected parents, we choose 1,047 plants that were the most closely related to each of the selected parents. This was accomplished through an iterative process for each parent that selected the genets with the highest value in **K** between each parent and the remaining ∼4,000 individuals. We also insured that each half‐sib family had a minimum of five genets in the validation population. To capture the diversity of TLI‐Cycle 7, we then selected 100 plants that had the lowest **K** values compared with the parent plants. Finally, a random subset of 231 genets were added to replace any individuals that had died. This resulted in a validation set with three unique subgroups—most closely related, most distantly related, and random subsets—with the validation population being weighted toward the selected parents. Principal component analysis of the marker matrix for these genets was used to verify these partitions.

The validation population individuals were transplanted to Jiffypots (Jiffy Products of America) sized 45 mm square and 55 mm deep to allow vigorous growth prior to field establishment. The TLI‐Cycle 7 validation population was randomly divided, with 600 of the plants being planted in a location with irrigation (internal field code KEB, 38°46′18.23″ N, 97°34′8.11″ W) and the remaining plants in a nonirrigated location (internal field code KOH, 38°46′21.84″ N, 97°35′31.76″ W). Plants were arranged randomly in the field locations, that is, plantings did not follow any known family structure, with each plant a unique genet with no replication or common check genet. These plants were transplanted during the last week of October 2017 and spaced on a uniform grid of 0.91‐m centers to allow for spatial correction of phenotypic data. Phenotypic evaluation was performed in 2018 and 2019. After the parents were intermated to produce TLI‐Cycle 8 seed in 2018, one ramet of each parent was placed in the field alongside each experimental location using the same planting design and phenotypically evaluated beginning in 2019.

### 2.4 TLI‐Cycle 8

TLI‐Cycle 8 seed was harvested from the 109 unique TLI‐Cycle 7 maternal genets that produced seed. Similar to TLI‐Cycle 7, 40–70 seeds from each half‐sib family were started in Jiffypots sized 45 mm square and 55 mm deep in early August 2018. Tissue collection for genotyping (genetic profiling) began in early September 2018, with ∼45 genets per each mother being sampled based on visual selection to remove seedlings with low vigor. In total, 5,130 genets were sampled for DNA extraction and GS.

Using the GEBVs and manual selection similar to previous cycles, 98 parents were selected to intermate to form the TLI‐Cycle 9 population. These parents were selected with GEBVs derived from the phenotypic data of TLI‐Cycle 6 and the TLI‐Cycle 7 validation population. In addition to using TLI‐Cycle 8 plants, five parents were selected from the TLI‐Cycle 7 validation population based on phenotypic performance and one additional plant was added because of its large seed size (identified in a separate evaluation) for a total of 104 parents. These genets were moved directly into the greenhouse and followed similar protocol for cloning, vernalizing, and intermating as TLI‐Cycle 7.

The TLI‐Cycle 8 validation population was made of plants randomly selected from among the genotyped individuals, after the selected parents had been removed, with a total of 1092 genets. Genets were divided between an irrigated (572 genets at KEB) and a nonirrigated site (520 genets at KOH) and transplanted in late October 2018. Similar to the TLI‐Cycle 7 validation population, there were no replications or check genets, and genets were randomly distributed on a uniform grid spacing of 0.91 m. After greenhouse intermating was complete, parent plants were placed in rows adjacent to the validation population in spring 2019. As these plants had already been cloned, one ramet from each parental genet was established in a randomized order in each of the two sites.

### 2.5 TLI‐Cycle 9

In early August 2019, the TLI‐Cycle 9 population was initiated by planting seed in Jiffypots sized 45 mm square and 55 mm deep. Similar to previous cycles, ∼50 seeds were started for each maternal half‐sib family, with visual selection for plants to genotype. In total, 4,174 genets were genotyped for GS. TLI‐Cycle 9 parents were formed by selecting 100 genets using GEBV developed from phenotypic data from TLI‐Cycle 6, 7, and 8, with 1,004 genets randomly selected to form the validation population. In October 2019, the validation population was planted with no replication or check genets on a 0.91‐m grid across an irrigated (504 genets at KEB) and a nonirrigated site (500 genets at KOH).

### 2.6 Genomic profiling

All genomic profiling was completed using genotyping‐by‐sequencing (GBS) with a two‐enzyme restriction digest as described by Poland et al. (2012). All genets were pooled into 192‐plex libraries that were sequenced on Illumina HiSeq machines at Hudson Alpha, Huntsville, AL. For TLI‐Cycle 7–9, there were often a large number of samples with limited read coverage. For TLI‐Cycle 7 genets with low sequencing data, plants were resampled with an additional 192‐plex run on an Illumina NextSeq, whereas TLI‐Cycle 8 resequencing protocol was similar to original runs.

Single nucleotide polymorphisms (SNPs) were called with the TASSEL GBSv2 pipeline (Glaubitz et al., 2014) using the draft genome assembly of *Thinopyrum intermedium* provided by the *Thinopyrum intermedium* Genome Sequencing Consortium (https://phytozome-next.jgi.doe.gov/info/Tintermedium_v2_1). For each cycle of prediction, (TLI‐Cycle 7, 8, 9) the GBS pipeline was run separately while including all samples from current and previous cycles using consistent filtering parameters across cycles. This allowed genetic variants to be continuously identified within each breeding cycle from changing allele frequencies and incorporation of novel germplasm (i.e. inclusion of a large seed size genet in TLI‐Cycle 8). Filtering consisted of the following: (a) only retaining 64‐bp tags that aligned to one location in the genome, thus preventing homoelogous loci from being combined; (b) sequencing depth >4 per loci per individual were required to call a homozygous genotype. If a SNP site had less than four reads, it was set to missing using a custom Perl script. Heterozygotes were called with a minimum of two contrasting tags per locus; (c) maximum missing data per marker was 70%; (d) SNPs with a minor allele frequency <0.01 were discarded; and (e) individuals that had >95% missing data were eliminated from further analysis. After filtering, remaining SNPs were imputed with Beagle version 4.1 using the default parameters (Browning & Browning, 2016). The number of SNPs before and after filtering and the number of individuals for each cycle are provided in Supplemental Table S1.

### 2.7 Phenotypic analysis

Across all years and cycles, all key traits for selection in addition to secondary traits were collected. The most important traits for the breeding program were free threshing, shattering, spike yield, and seed mass; other traits that were recorded and the range of values are listed in Supplemental Table S2. Analysis was completed for each cycle and trait to evaluate the effectiveness of the GS selection and to update the GS models for future prediction cycles.

For all cycles, a mixed model was used to develop BLUPs for each genet using ASREML version 4.1 (Gilmour et al., 2015), with BLUP denoting output from the mixed model for observed phenotypic data. The model accounted for spatial effects using a row–column autoregressive order 1 (AR1 × AR1) model of residual variance, relatedness among genets using the genomic relationship matrix, and random effects for male, female, and parent interaction. This model, or minor variations, has previously been used by TLI within the breeding program (Crain et al., 2020a, 2021; DeHaan et al., 2018). The general form of the model is as follows (Isik et al., 2017):

(1) y=Xb+Zu+e

Where **y** is a vector of observed trait, **X** and **Z** are incidence matrix for fixed and random effects that relate observed trait values to genets, **b** and **u** are vectors of effect estimates for fixed and random model components, and **e** is a vector of random residuals. The vector **y** is normally distributed with mean **Xb** and variance **V**, y∼N(Xb,V). The total variance, **V** can be represented as $V=(\begin{array}{c} u\\ e \end{array})=(\begin{array}{cc} G & 0\\ 0 & R \end{array})$, where **G** is the variance of random genets using the genomic relationship matrix, and **R** is the residual variance using the row‐column design. The **G** component can further be defined as **G** = $\sigma_{A}^{2}$ **K**, where **K** is the realized additive genomic relationship matrix (genomic prediction) and $\sigma_{A}^{2}$ is the additive genetic variance. The **R** structure is separately defined for each experiment site as **R =** $\sigma_{e}^{2}\Sigma_{c}\left(\rho_{c}\right)⊗\Sigma_{r}\left(\rho_{r}\right)$ where $\sigma_{e}^{2}$ is the residual error variance that is normally and independently distributed for each site, and $\Sigma_{c}\left(\rho_{c}\right)$ is the matrix of the column (row) correlation.

Using the mixed model, we assessed TLI‐Cycle 7 and 8 independently, with **R** comprised of two sites for the irrigated and nonirrigated site. The BLUPs were compared with the GEBVs to assess GS prediction accuracy. The GS training population reflected the data that had been observed and was updated accordingly. For TLI‐Cycle 7 prediction, the only training data was TLI‐Cycle 6, which comprised one site location. For TLI‐Cycle 8 predictions, the **R** structure included three factors (TLI‐Cycle 6, TLI‐Cycle 7—KEB and KOH—where KEB and KOH are identifies of irrigated and nonirrigatyed sates), increasing to five separate factors for TLI‐Cycle 9 prediction (TLI‐Cycle 6, TLI‐Cycle 7—KEB and KOH—and TLI‐Cycle 8—KEB and KOH).

Equation 1 also provided variance ratios to compute narrow‐sense heritability (*h* ^2^) as follows (Jordan et al., 1999):

(2) $$h^{2}=\frac{\sigma_{\mathrm{additive}}^{2}}{\sigma_{\mathrm{phenotypic}}^{2}}\;$$

where

(3) $$\;\sigma_{\mathrm{phenotypic}}^{2}=\;\sigma_{a}^{2}+\;\sigma_{m}^{2}+\;\sigma_{f}^{2}+\;\sigma_{m\times f}^{2}+\sigma_{a\;\times \;\mathrm{site}}^{2}\;\sigma_{\varepsilon }^{2}$$

and $\sigma_{\text{phenotypic}}^{2}$ is the total variance; $\sigma_{a}^{2}$ is the additive genetic, or breeding value variance (Falconer & Mackay, 1996); $\sigma_{m}^{2}$, $\sigma_{f}^{2}$, and $\sigma_{m\times f}^{2}$, is the nonadditive variance attributable to mother, father, and mother × father interaction, respectively (Lynch & Walsh, 1998; Isik et al., 2017; Mrode, 2005), $\sigma_{a\times site}^{2}$is the genet (genotype) × site interaction, and $\sigma_{\varepsilon }^{2}$ is the residual error variance.

In addition to evaluating GS effectiveness and updating the GS model, we also evaluated TLI‐Cycle 7 data within each year and trait to examine the magnitude of change in the breeding program through GS. As the TLI‐Cycle 7 validation population had distinct subsets, we used a linear model to determine if these subsets varied based on the effect of selection. We used GEBVs (genomic prediction) that were previously predicted from the GS models to choose the highest performing 150 individuals for each trait to compare with the distantly related and random subsets. Choosing the top 150 individual allowed for this group to most closely mirror the parent population as adding more individuals would result in a more divergent group from the parents. In 2019, the genets selected to form the TLI‐Cycle 8 generation were also evaluated in the field, providing an opportunity to estimate the magnitude of selection within the population. This allowed for two distinct groups from the same cycle—selected parents and unselected validation population—to be compared to determine the realized selection differential, the difference between the two groups. For TLI‐Cycle 7 we were able to both calculate the realized selection differential and the expected differential based on GEBVs. For both determining TLI‐Cycle 7 validation population subset effect and the realized selection differential, we used the following linear model:

(4) $$y_{ij}=\;u+\alpha_{i}+\beta_{j}+{\left(\alpha \beta \right)}_{ij}+\;e_{ij}$$

where *y_ij_* is the observed trait value of the *i*th group and *j*th location, *u* is the overall mean, α is the fixed group effect (either TLI‐Cycle 7 subset, or parent–nonparent for each model respectively), β is the fixed effect of experimental site, (αβ)*_ij_* is the interaction between factors *i* and *j*, and *e_ij_* is the residual error. Multiple comparison for group effects were made with Fisher's LSD using the *agricolae* R package (de Mendibury, 2020). This model was chosen as the placement of selected parents were not randomized, on the edge of sites, within the TLI‐Cycle 7 validation population and the AR1 × AR1 model would have been inappropriately biased. For the comparison of parents and nonparents, a Wald test was used to determine if the group effect was significant.

The breeders equation (Falconer & Mackay, 1996):

(5) $$R\;=\;Sh^{2}$$

was used to estimate the genetic gain achieved through GS selections as well predict potential genetic gain from using GS within the breeding program. In Equation 5, *R* is the response to selection, *S* is the selection differential, and *h* ^2^ is the narrow‐sense heritability. Dividing the response to selection by years to complete the breeding cycle results in genetic gain per year.

### 2.8 Genomic prediction

All genomic predictions, GEBVs (Calus, 2010), were completed using the *rrBLUP* package (Endelman, 2011). The prediction model has the following form:

(6) y=Wg+e

where **y** is a vector of BLUPs calculated from Equation 1, **W** is a matrix relating genets to BLUPs, **g** is a vector of genotypic values for each genet, and **e** is a vector of random residuals. The vector **g** is distributed as **g** ∼ N(0,**K** $\sigma_{g}^{2}$), where **K** is the realized additive relationship matrix calculated according to Endelman and Jannink (2012) and $\sigma_{g}^{2}$ is the additive genotypic variance; **K** is computed as θ**MM'** where θ is a proportionality constant and **M** is the genotype matrix with dimension of *n* rows of individuals and *m* columns of markers. For each cycle, the model was trained on all previous data for year of phenotypic observation, for example, free‐threshing Year 1 collected from TLI‐Cycle 6 and 7 was used to predict free‐threshing Year 1 for TLI‐Cycle 8, with predictions being made on the seedling genets that had been genotyped. Pearson correlations and confidence intervals between GEBVs and BLUPs for traits were calculated using the *psychometric* R package (Fletcher, 2010)

### 2.9 Data availability

The genotypic datasets are available at NCBI sequence read archive (https://www.ncbi.nlm.nih.gov/bioproject/) as part of the umbrella BioProject PRJNA609325. All phenotypic data and the scripts created for data analysis have been placed in the Dryad Digital Repository (https://doi.org/10.5061/dryad.zw3r2285n).

## 3 RESULTS

Beginning in 2017, TLI implemented a GS program, completing one cycle of selection per year. Ideally, this method will increase the rate of genetic gain and accelerating domestication and improvement of this new perennial grain crop. Using data from 2017 through 2019, we evaluated three lines of evidence to evaluate the effectiveness of GS. First, we examined the correlation between forward GS predictions and the observed value from evaluation in subsequent years. Second, we investigated the established subgroups of the validation population testing if individuals most closely related to the selected parents had superior performance. Finally, using the perennial nature of IWG, we evaluated the superiority of selected parents per se compared with unselected populations. Using this information, we calculated the realized selection differential and the expected genetic gain from GS.

### 3.1 Correlation between GEBV and observed phenotypes

**FIGURE 1:**
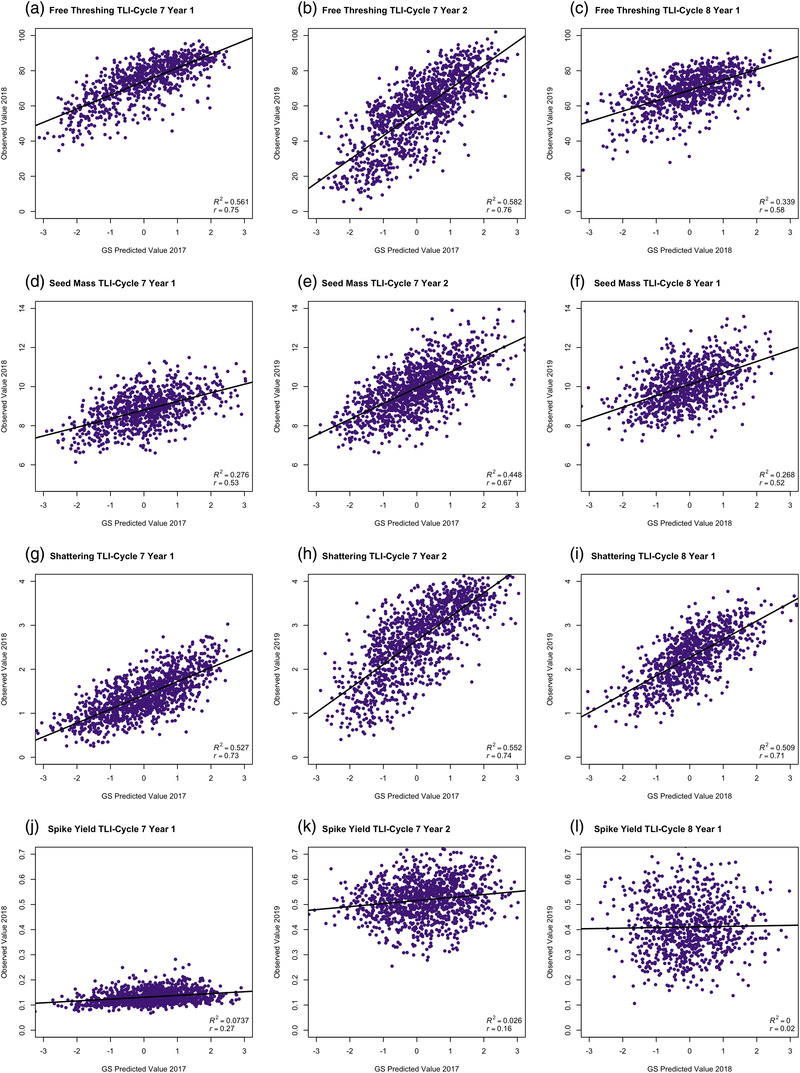
(a–l) Panels represent the relationship between normalized genomic estimated breeding value (*x* axis) and best linear unbiased predictor value (*y* axis) for each trait. Each panel represents one trait year combination for the TLI‐Cycle 7 or TLI‐Cycle 8 intermediate wheatgrass breeding program. Genomic prediction for TLI‐Cycle 7 were completed in 2017 with phenotypic observations in 2018 and 2019. TLI‐Cycle 8 predictions were made in 2018 with phenotypic observations in 2019. Panels include line of best fit and correlation for each trait

Genomic selection was used to make forward predictions, and observed phenotypes in the following years were generally significantly related to predicted values. For the TLI‐Cycle 7 validation population, we predicted 32 unique trait–year combinations across two separate years (Table 1). Because of the perennial crop cycle, we modeled and predicted each year of observation—the first or second year in the field for each cohort—separately for each trait. For the TLI‐Cycle 8 validation population, there have been 14 traits evaluated in 1 yr, 2019. The correlation between GEBVs and the phenotypic BLUPs for each of the 46 traits ranged from 0.02 to 0.76 (Table 1). The priority traits—free threshing, shattering, and seed mass—had high prediction accuracy with all accuracy >0.52, yet spike yield had the lowest observed accuracies ranging from 0.02 to 0.27 (Figure 1). The high prediction accuracy for these traits was consistent both across years and cycles. Spike yield had low prediction accuracies across both years and cycles of observations; additionally, spike yield was consistently one of the most difficult traits to predict. There was also a significant trend (*r* = .57, *p* < .001) that prediction accuracy was positively correlated with heritability.

**TABLE 1:** Relationship between predicted genomic estimated breeding values and best linear unbiased predictor values for TLI‐Cycle 7 and TLI‐Cycle 8 traits collected over two years, 2018 and 2019. Table includes the number of observations, 95% confidence interval for correlation values, and narrow‐sense heritability (*h* ^2^) for each observed trait year and cycle combination. Priority traits are in bold

Cycle	Year	Trait	*n*	Correlation between predicted GEBV and observed BLUP	95% confidence interval	*h* ^2^
**TLI‐Cycle 7**	**2018**	**Free threshing (%)**	**851**	**0.75**	**0.72–0.78**	**0.77**
**TLI‐Cycle 7**	**2019**	**Free threshing (%)**	**1165**	**0.76**	**0.74–0.79**	**0.75**
**TLI‐Cycle 8**	**2019**	**Free threshing (%)**	**870**	**0.58**	**0.54–0.63**	**0.83**
TLI‐Cycle 7	2018	Maturity	1157	0.54	0.49–0.58	0.64
TLI‐Cycle 7	2018	Number of florets per spike	1140	0.13	0.07–0.19	0.43
TLI‐Cycle 7	2019	Number of florets per spike	1168	0.41	0.36–0.46	0.57
TLI‐Cycle 8	2019	Number of florets per spike	871	0.42	0.36–0.47	0.24
TLI‐Cycle 7	2018	Number of florets per spikelet	1140	0.02	−0.04 to 0.08	0.39
TLI‐Cycle 7	2019	Number of florets per spikelet	1168	0.34	0.28–0.39	0.56
TLI‐Cycle 8	2019	Number of florets per spikelet	871	0.41	0.35–0.46	0.77
TLI‐Cycle 7	2018	Percentage seed set	1134	0.55	0.51–0.59	0.52
TLI‐Cycle 7	2019	Percentage seed set	1162	0.32	0.26–0.37	0.32
TLI‐Cycle 8	2019	Percentage seed set	862	0.23	0.17–0.29	0.44
TLI‐Cycle 7	2018	Plant height (cm)	1152	0.50	0.46–0.54	0.47
TLI‐Cycle 7	2019	Plant height (cm)	1176	0.18	0.12–0.23	0.48
TLI‐Cycle 8	2019	Plant height (cm)t	959	0.47	0.42–0.52	0.41
TLI‐Cycle 7	2018	Seed area (mm^2^)	851	0.58	0.53–0.62	0.66
TLI‐Cycle 7	2019	Seed area (mm^2^)	1165	0.67	0.64–0.70	0.59
TLI‐Cycle 8	2019	Seed area (mm^2^)	865	0.53	0.48–0.58	0.52
TLI‐Cycle 7	2018	Seed density	848	0.34	0.28–0.40	0.46
TLI‐Cycle 7	2019	Seed density	1163	0.52	0.47–0.56	0.56
TLI‐Cycle 8	2019	Seed density	865	0.52	0.47–0.57	0.60
TLI‐Cycle 7	2018	Seed length (mm)	851	0.66	0.62–0.69	0.04
TLI‐Cycle 7	2019	Seed length (mm)	1165	0.70	0.67–0.73	0.03
TLI‐Cycle 8	2019	Seed length (mm)	865	0.53	0.48–0.58	0.72
**TLI‐Cycle 7**	**2018**	**Seed mass (mg)**	**848**	**0.53**	**0.48–0.57**	**0.51**
**TLI‐Cycle 7**	**2019**	**Seed mass (mg)**	**1163**	**0.67**	**0.64–0.70**	**0.52**
**TLI‐Cycle 8**	**2019**	**Seed mass (mg)**	**865**	**0.52**	**0.47–0.57**	**0.80**
TLI‐Cycle 7	2018	Seed width (mm)	851	0.51	0.46–0.56	0.09
TLI‐Cycle 7	2019	Seed width (mm)	1165	0.61	0.57–0.64	0.01
TLI‐Cycle 8	2019	Seed width (mm)	865	0.61	0.56–0.65	0.40
TLI‐Cycle 7	2018	Seeds per spike	1135	0.49	0.44–0.53	0.43
TLI‐Cycle 7	2019	Seeds per spike	1163	0.24	0.18–0.29	0.26
TLI‐Cycle 8	2019	Seeds per spike	862	0.18	0.12–0.24	0.62
**TLI‐Cycle 7**	**2018**	**Shattering**	**1141**	**0.73**	**0.70–0.75**	**0.53**
**TLI‐Cycle 7**	**2019**	**Shattering**	**1168**	**0.74**	**0.72–0.77**	**0.60**
**TLI‐Cycle 8**	**2019**	**Shattering**	**871**	**0.71**	**0.68–0.74**	**0.59**
TLI‐Cycle 7	2018	Spike length (cm)	1142	0.51	0.47–0.56	0.54
**TLI‐Cycle 7**	**2018**	**Spike yield (g)**	**1141**	**0.27**	**0.22–0.33**	**0.41**
**TLI‐Cycle 7**	**2019**	**Spike yield (g)**	**1169**	**0.16**	**0.11–0.22**	**0.32**
**TLI‐Cycle 8**	**2019**	**Spike yield (g)**	**868**	**0.02**	**−0.05 to 0.09**	**0.66**
TLI‐Cycle 7	2018	Spikelets per inflorescence	1140	0.50	0.46–0.54	0.24
TLI‐Cycle 7	2019	Spikelets per inflorescence	1169	0.42	0.37–0.47	0.35
TLI‐Cycle 8	2019	Spikelets per inflorescence	871	0.35	0.29–0.41	0.26
TLI‐Cycle 7	2018	Stem angle	1180	0.55	0.51–0.59	0.55
TLI‐Cycle 7	2018	Stem strength	997	0.42	0.37–0.47	0.56

### 3.2 Effect of selected subgroups within validation population

**FIGURE 2:**
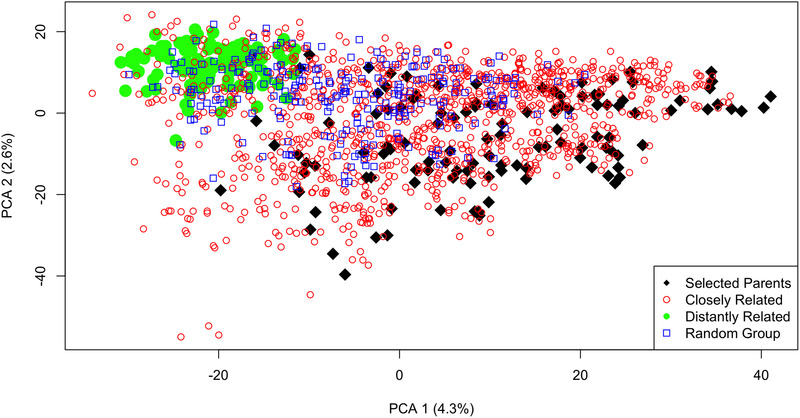
Scatterplot of the first (*x* axis) and second (*y* axis) principal components (PCs) for the TLI‐Cycle 7 selected individuals. Individual genets are plotted by color and symbol to their respective group. Total variance explained by each PC is listed in the axes

The TLI‐Cycle 7 validation population was selected with three distinct subgroups: genets closely related to the selected parents (closely related), genets distantly related (distantly related), and a random subset (Figure 2). This partitioning provided a way to test if GS could identify superior genets and effectively shift the population mean. The closely related group was significantly superior to the distantly related group for free threshing, shattering, and seed mass, which are under strong selection within the breeding program. The random additions were also inferior to the closely related group for these traits (Table 2) suggesting that the GS model was correctly predicting genet performance and driving selection.

**TABLE 2:** TLI‐Cycle 7 validation population traits observed over 2 yr, 2018 and 2019. The validation population was structured into three groups that were closely related, distantly related, and a random group compared with the selected parents. For each trait, the mean for each group is reported and groups having different letter codes are statistically different using Fisher's LSD at *p* < .05. Priority traits are in bold

Year	Trait	Distantly related	Closely related	Random
2019	Brittle rachis	0.31a	0.29a	0.28a
**2018**	**Free threshing**	**52.37c**	**86.43a**	**74.25b**
**2019**	**Free threshing**	**30.53c**	**79.04a**	**49.77b**
2019	Lodging	6.40a	6.05a	5.78a
2018	Maturity	58.37a	59.42a	55.55b
2019	Maturity	64.80a	64.85a	65.25a
2018	Number of florets per spike	200.74ab	204.93a	189.54b
2019	Number of florets per spike	147.01b	170.43a	153.93b
2018	Number of florets per spikelet	9.22a	8.99a	9.04a
2019	Number of florets per spikelet	6.58a	6.83a	6.59a
2019	Peduncle width	9.77a	9.74a	9.95a
2018	Percentage seed set	0.10a	0.11a	0.05b
2019	Percentage seed set	0.38a	0.38a	0.39a
2018	Plant height	87.97b	93.37a	81.36c
2019	Plant height	136.28a	139.33a	136.81a
2018	Seed area	8.38b	9.16a	8.31b
2019	Seed area	7.59c	8.83a	8.03b
2018	Seed density	1.28c	1.43a	1.36b
2019	Seed density	1.41c	1.65a	1.52b
2018	Seed length	6.3b	6.83a	6.24b
2019	Seed length	6.29c	7.05a	6.49b
**2018**	**Seed mass**	**8.11b**	**9.79a**	**8.54b**
**2019**	**Seed mass**	**8.91c**	**11.57a**	**9.89b**
2018	Seed width	1.72b	1.76a	1.71b
2019	Seed width	1.57b	1.65a	1.59b
2018	Seeds per spike	19.76a	18.01a	9.86b
2019	Seeds per spike	53.26a	56.39a	56.11a
**2018**	**Shattering**	**2.16a**	**0.77c**	**1.50b**
**2019**	**Shattering**	**3.39a**	**1.32c**	**3.13b**
2018	Spike emergence	0.77a	0.65b	0.5c
2018	Spike emergence	23.52a	20.05b	14.69c
2018	Spike length	32.06b	36.51a	31.89b
**2018**	**Spike yield**	**0.17a**	**0.14a**	**0.09b**
**2019**	**Spike yield**	**0.46b**	**0.54a**	**0.54a**
2018	Spikelets per inflorescence	21.78b	23.59a	20.93c
2019	Spikelets per inflorescence	22.07c	24.68a	23.26b
2018	Stem angle	44.45c	64.87a	51.32b
2018	Stem strength	1616.84b	1787.38a	1473.07c
2019	Stem strength	879.63a	896.25a	928.69a

For spike yield, there were no significant differences between the close and distantly related groups in 2018, with the closely related group being significantly better than the distantly related group in 2019. Surprisingly, the random group performed as well as the most closely related group in 2019, yet in 2018, this group was significantly worse than the closely related group (Table 2). For traits that had no direct selection pressure, such as seeds per head, lodging, and percentage fertility, there were no differences between the distantly and closely related groups, indicating minimal genetic correlation and limited impact of indirect selection on other traits. Several traits, however, did have significant differences such as seed length and seed width. These traits were not direct selection targets but maybe have been indirectly a result of correlations between those traits and the priority traits of seed mass and grain yield. The partitioned TLI‐Cycle 7 validation population showed that GS was shifting trait phenotypes according to selection preferences.

### 3.3 Realized selection differential

**FIGURE 3:**
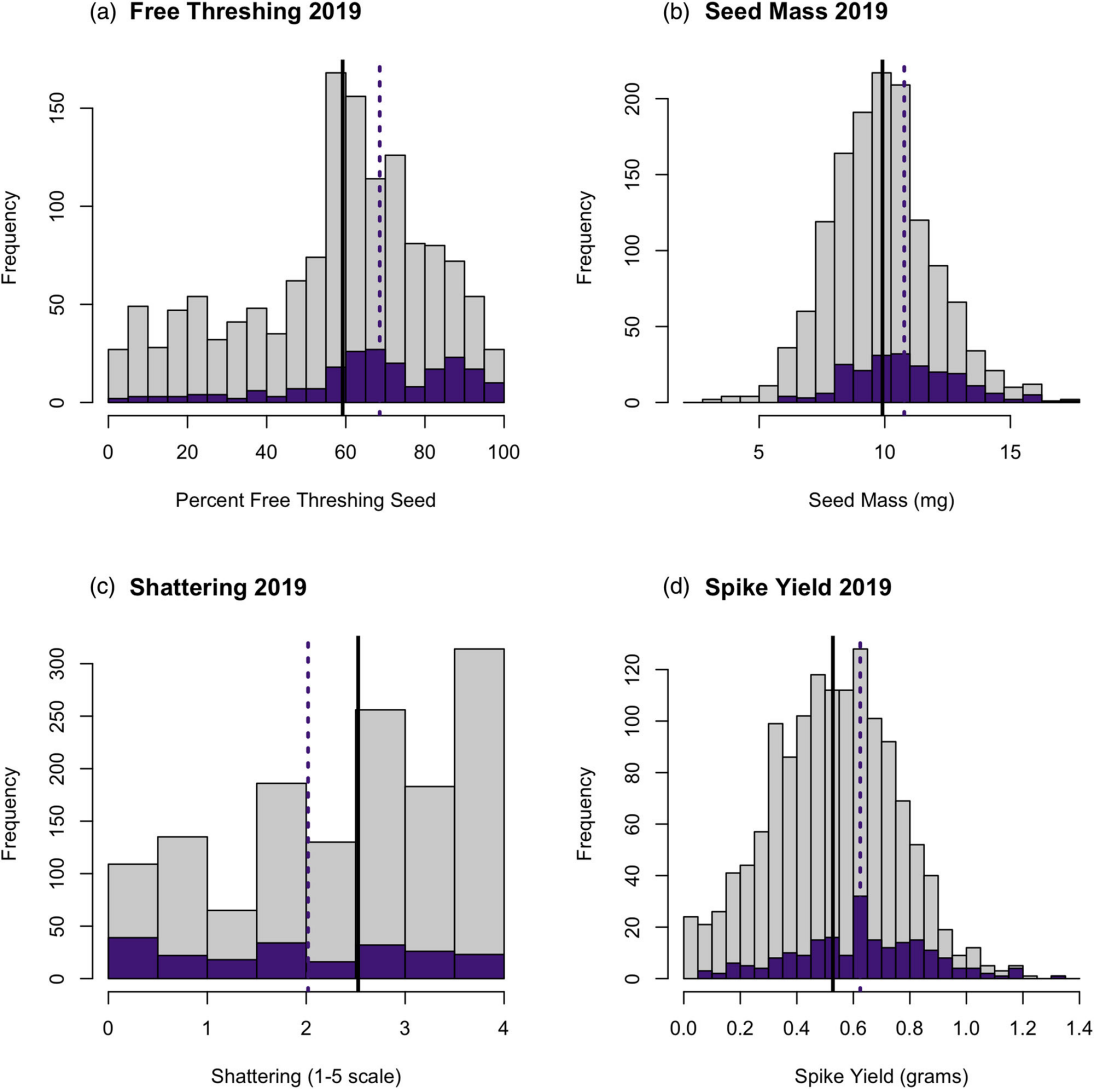
Histograms of trait distribution for TLI‐Cycle 7 genets in 2019. Each panel represents one trait with the validation population shown with black histogram. The purple colored bars represent the parent distribution for the same trait. Vertical solid line is the mean of the validation population, with purple dashed line representing the mean of the parents

Leveraging the perennial nature of IWG, the TLI‐Cycle 7 selected parents were placed in the TLI‐Cycle 7 validation population fields after intermating to evaluate if the GS selected parent genets had superior performance to nonselected genets. In 2019, we observed trait values for the selected parents compared with the validation population (Table 3) and calculated the mean between these two groups as the realized selection differential. These realized selection differentials were also compared with the differences in GEBVs for the selected parents and the validation populations. For nearly all traits, there was a significance difference between the selected parents and validation population. For priority traits the realized selection differentials were 19% higher for free threshing, 23% less for shattering, 11% higher for seed weight, and 22% higher for spike yield (Figure 3). Traits not targeted by selection, such as plant height and maturity, showed no difference between the groups. Overall, the expected selection differential, based solely on GS predicted values, and the observed selection differentials were highly correlated (*r =* .69, *p* = .007) providing evidence that the models were accurately predicting plant phenotypes and enabling GS.

**TABLE 3:** TLI‐Cycle 7 traits measured in 2019 for two groups: validation population and the selected parents. The mean for each group is reported along with *p* value for group effect in the model. Realized selection differential, difference between the observed parent and validation population has been used to calculate response to selection. Expected selection differential is the selection differential based on genomic selection predictions and provided for comparison between expected and observed values. Expected response to selection is calculated from realized selection differential and trait heritability. Priority traits are in bold

Trait	*p* value	Parent population mean	Validation population mean	Realized selection differential	Realized selection differential	Expected difference GEBV^a^	Expected selection differential	Expected response to selection units	Expected response to selection
					%		%		%
Brittle rachis	0.036	0.25	0.32	−0.07	−22	NP^b^	–	−0.01	−3
**Free threshing**	**<.001**	**68.57**	**57.52**	**11.05**	**19**	**10.13**	**32**	**8.29**	**14**
Lodging	<.001	4.90	6.18	−1.28	−21	–	–	−0.10	−2
Maturity	0.609	65.03	64.85	0.18	0	–	–	0.06	0
Number of florets per spike	<.001	187.42	158.27	29.15	18	6.02	4	16.62	11
Number of florets per spikelet	<.001	7.68	6.78	0.90	13	0.17	3	0.50	7
Peduncle width	<.001	10.73	9.90	0.83	8	–	–	0.42	4
Percentage seed set	0.052	0.33	0.35	−0.02	−6	0.01	2	−0.01	−3
Plant height	0.772	136.31	135.97	0.34	0	1.26	1	0.16	0
Seed area	<.001	8.31	7.88	0.43	5	0.18	2	0.25	3
Seed density	<.001	1.59	1.49	0.10	7	0.01	1	0.06	4
Seed length	<.001	6.77	6.51	0.26	4	0.14	2	0.01	0
**Seed mass**	**<.001**	**10.78**	**9.75**	**1.03**	**11**	**0.25**	**3**	**0.54**	**6**
Seed per spike	<.001	58.77	52.87	5.90	11	3.44	10	1.53	3
Seed width	0.006	1.59	1.57	0.02	1	0.00	0	0.00	0
**Shattering**	**<.001**	**2.02**	**2.62**	**−0.60**	**−23**	**−0.21**	**−9**	**−0.36**	**−14**
**Spike yield**	**<.001**	**0.62**	**0.51**	**0.11**	**22**	**0.03**	**11**	**0.04**	**8**
Spikelets per Inflorescence	<.001	24.40	23.27	1.13	5	0.39	2	0.40	2
Stem strength	<.001	1276.19	905.37	370.82	41	–	–	77.87	9

^a^GEBV, genomic estimated breeding value.

^b^NP, trait was not predicted in genomic selection models.

### 3.4 Expected genetic gain

We calculated the expected genetic gain for 19 phenotypic traits using the realized selection differential (Table 3). Priority traits under selection were expected to show gains of eight percentage points for free threshing, 0.36 units for less shattering, 0.54 mg higher seed mass, and 0.04 g increase in spike yield. Traits under no selection showed very little movement from the population mean. For example, plant height is expected to increase 0.16 cm and had a nonsignificant difference of only 0.34 cm even with high heritability of the trait (*h^2^* = .48). These expected gains indicate that GS can be used to rapidly improve IWG traits.

## 4 DISCUSSION

### 4.1 TLI breeding pipeline

**FIGURE 4:**
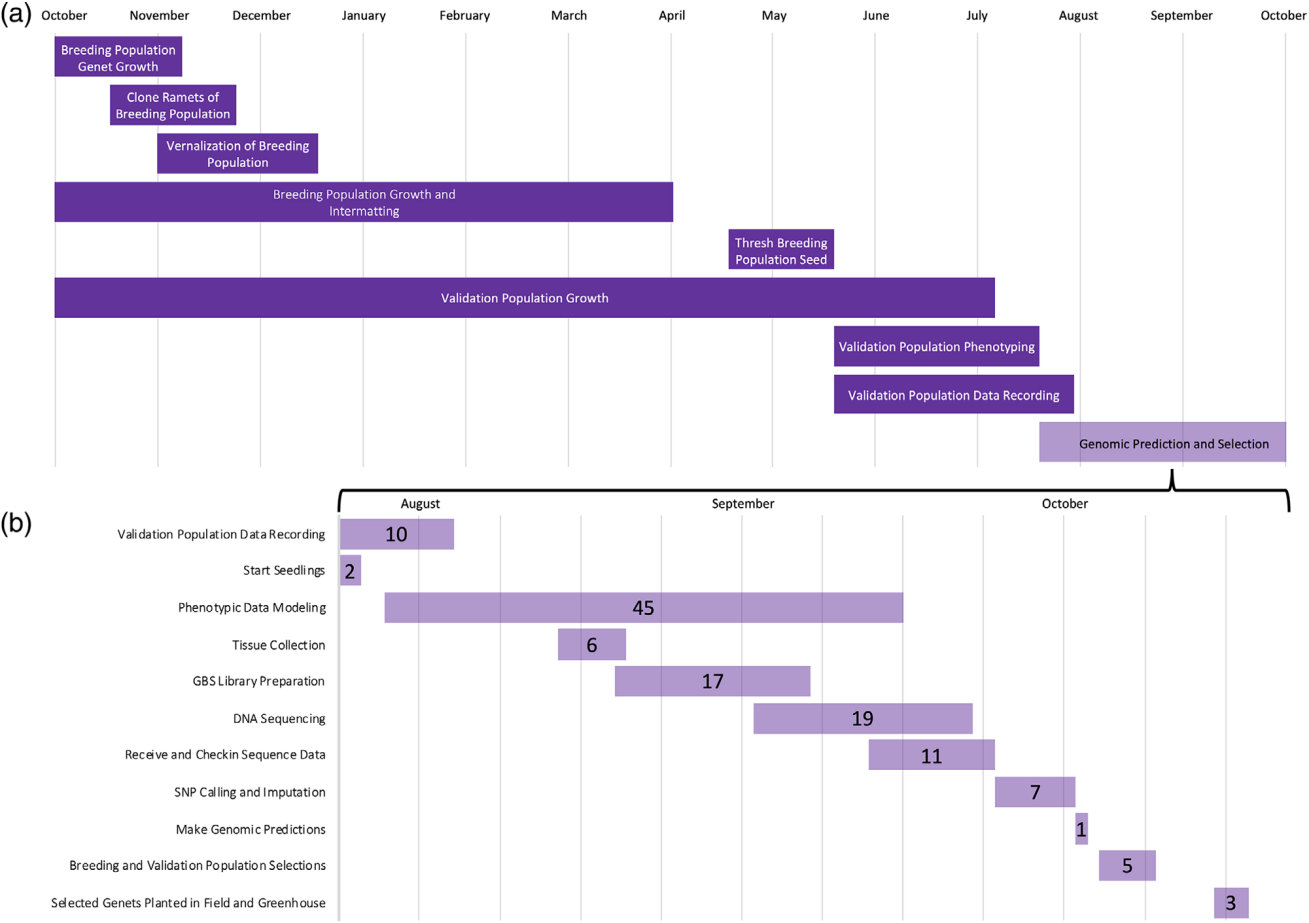
Gantt chart of intermediate wheatgrass breeding program. (a) Panel diagrams activities occurring within the 1‐yr breeding cycle beginning in early October each year. Breeding population refers to the ∼100 selected parents that are intermated in the greenhouse to form seed for the following cycle. The validation population are field‐grown plants with phenotypic evaluation that are used to train genomic selection (GS) models. Activities are labeled within their respective timeframe. (b) Panel diagrams the specific steps during the GS with each activity in the program listed on the *y* axis with approximate dates of each activity and length of activity in the *x* axis and designated by approximant number of days inside the box. This basic outline has been followed each year for the previous three intermediate wheatgrass breeding cycles at The Land Institute, Salina, KS

The TLI IWG breeding program has now completed three cycles of selection in 3 yr using GS to select parents and validation populations from ∼4,000 genets each cycle. Since 2017, ∼40,000 phenotypic data points have been collected each year to refine the GS models. Logistically, there are several issues that programs transitioning to GS should be ready to address to complete the entire breeding cycle in a defined timeline (Figure 4). Running a GS program requires completing both phenotypic and genotypic analyses simultaneously. Often, as data is being collected in the field from validation populations in late summer, the next cycle of selection has to be started in the greenhouse to allow sufficient time for all genotyping to be completed. Additionally, as more data is collected, the statistical models, using both genomic and spatial information, take longer to complete. During the breeding cycle, the statistical models to generate GS training BLUPs have already been developed and are only ran to add the newest data. Any new model development, and associated time delay, occurs outside the short window (90 d) of making selections and advancements in the breeding cycle. The genotyping timeframe is constrained by having a late October planting deadline to ensure plants moved to the field can grow sufficiently to survive the winter. To efficiently process the volume of samples from DNA extraction to sequencing, data is processed in batches often resulting in the first sequencing data being received from core facilities before the final GBS libraries are completed. From starting genets in the greenhouse until final selections are planted in the field or greenhouse, the timeframe that the IWG breeding program has to complete genotyping, quality control, genomic predictions, selections, and transplanting is 90 d. Efficient data management and processing pipelines are required to quickly and carefully process the millions of datapoints generated from sequencing and the thousands of phenotypic data points that must be analyzed within the GS pipeline. While Zhang et al. (2016) propose a 2‐yr breeding cycle for GS within IWG, this slower rate directly impedes the rate of genetic gain compared with completing one breeding cycle per year. The distinct advantage of GS can be realized specifically when cycle time is reduced.

### 4.2 Effectiveness of GS

Numerous studies predict that GS can be used to increase the rate of genetic gain in IWG (Bajgain et al., 2020; Crain et al., 2020a; b; Zhang et al., 2016). Here we have examined the results of multiple cycles of GS implemented in an IWG breeding program. Overall, we found that correlations between predicted values and future observed values were generally quite high, with the main exception being spike yield. The low prediction value for spike yield could be due to the amount of environmental variance and genotype‐by‐environment that impact this trait from year to year. Crain et al. (2020a) showed similar results that traits with less genotype‐by‐environment interaction—free threshing and shattering—had high predictive abilities, while spike yield had low predictive ability. The GS models used to predict spike yield were built on data from 2016–2018, which were often dry years, but 2019 was an exceptionally wet year, potentially leading high genotype‐by‐environment interaction and severely reducing the prediction accuracy. Even though the GS predictions were low for spike yield, we observed a larger realized selection differential. This likely is due to more selection pressure being applied relative to other traits such as seed mass. As more years of data are collected, we anticipate that the models will improve as data from normal and extreme years are aggregated and the program will be better able to predict long‐term average performance rather than performance in a small number of specific environments.

Using the structure of the TLI‐Cycle 7 validation population and parents, we observed that traits are responding to selection. Expected yearly (cycle) genetic gains in priority traits were 14% increase in free threshing, 14% decrease in shattering, 6% increase in seed mass, and 8% increase in spike yield. One caveat is that these values were estimated from the validation population, which is not a truly random set of TLI‐Cycle 7 genets. Because the validation population was comprised of individuals with above‐average similarity to the selected plants, these estimated gains are conservative.

Evaluating numerous traits allows a better understanding of the component traits for selection target of grain yield. While spike yield is predicted to increase, we also noted that percentage seed set actually decreased in TLI‐Cycle 7 parent populations (Table 3). This also corresponded to an increase in the number of spikelets per inflorescence, florets per spike, and spikelets per spike. These data suggest that increased spike yield has resulted from increased total floret numbers rather than increased floret site utilization, which was the breeding target and desired outcome. Previous research in perennial ryegrass (*Lolium perenne* L.) has shown that increased seed yield can be attributed to a higher percentage seed set (Marshall & Wilkins, 2003). An increase in percentage seed set would be desirable so as not to develop varieties with larger heads, longer development times, and greater lodging tendency. Our results indicate that the easiest path to increased seed yield may be through undesirably large heads, so other approaches may be required to develop varieties with consistently higher seed set in diverse environments and supraoptimal temperatures.

Previously, TLI has completed a cycle of phenotypic selection in 2 yr, with the goal of achieving 10% or more gain per cycle. With the distinct advantage of GS enabling reduced breeding cycle time, the gains from TLI‐Cycle 7 projected over 2 yr (e.g. two cycles of GS) would be a 30% increase in free threshing, 30% decrease in shattering, 12% increase in seed mass, and 17% increase in spike yield, allowing GS to potentially perform significantly better than phenotypic selection. Crain et al. (2021) estimated that genetic gains per year using GS could be 3.8, 5.3, 6.6, and 2.6‐fold improvement for free threshing, shattering, seed mass, and spike yield over phenotypic selection. Our observed results were lower with only a threefold improvement expected in free threshing and shattering, 1.2‐fold increase in seed mass, and 1.7‐fold increase in spike yield. These genetic improvement estimates were made using one cycle (location) of plants with cross‐fold validation strategy, so these estimates represent an upper boundary on what is achievable per cycle without the confounding effects of genotype‐by‐environment across years and locations (Crain et al., 2020b). Additionally, these estimates assume selection on a single trait, while TLI currently selects for broad improvement of priority traits. While realized gains could be influenced by a variety of factors, such as the selection intensity placed on specific traits, measurement error and precision, and environmental variance, the current results appear to support strong genetic improvement through application of GS.

### 4.3 Future directions

**FIGURE 5:**
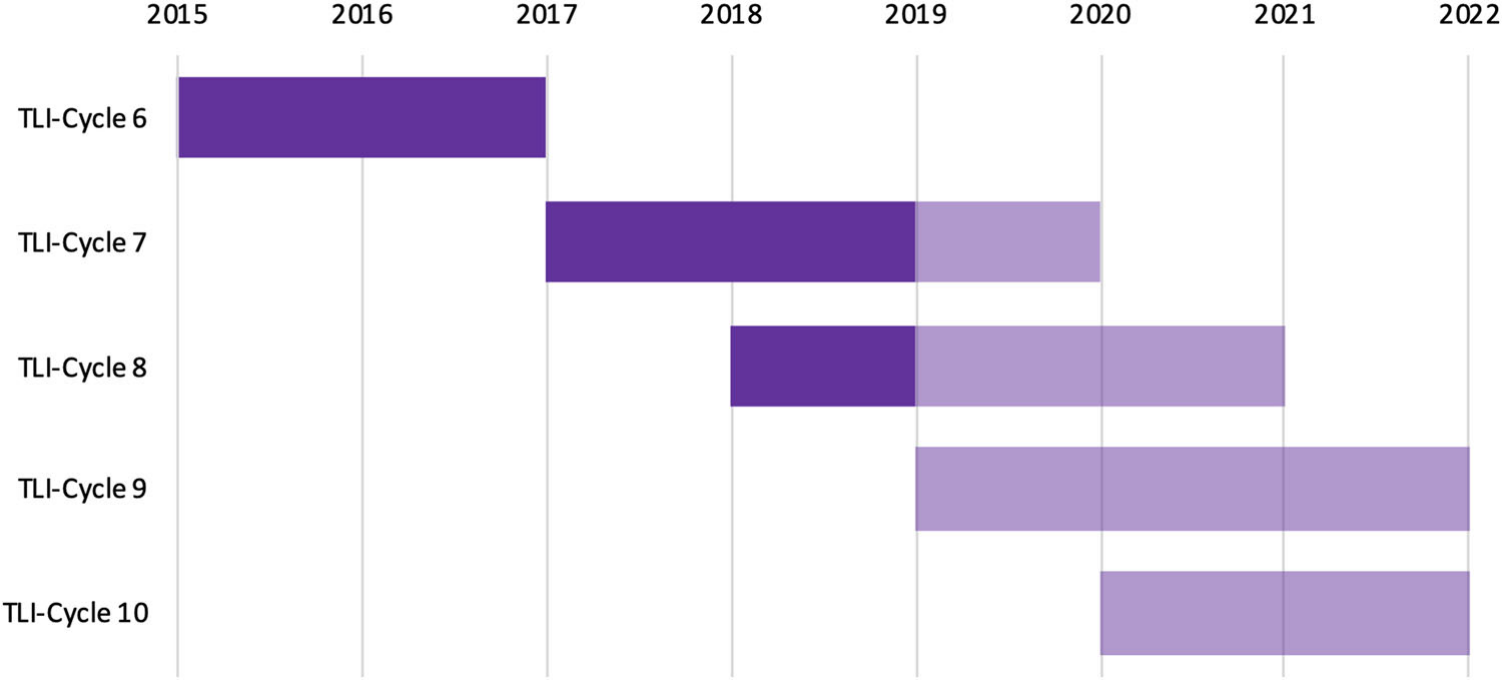
Diagram of The Land Institute (Salina, KS) intermediate wheatgrass genomic selection breeding program showing the duration of field evaluation for the training populations. Cycles are listed on the *y* axis with years of phenotypic collection on the *x* axis. Dark shading is completed and anticipated data collection is in light

Currently, GS appears very effective within the IWG breeding program. Using GS, TLI has completed one cycle of selection per year compared with a minimum of 2 yr per cycle with phenotypic selection. The current implementation of GS is very flexible, as data are still being collected on TLI‐Cycle 7 (Figure 5). By completing a selection cycle each year, but observing plants for up to three or more years, the breeding program is constantly making new genetic combinations, while also building a training population that truly reflects performance of IWG throughout a multi‐year lifespan. As a third or subsequent years of phenotypic observations are completed, models for long‐term traits, such as sustained yield, can be developed and used. The result will be a training population that may be two or more cycles behind the generation of selection. Further flexibility is provided by having multiple cycles of training populations, and if an outstanding genet is identified that was not in the crossing block, it can be added in future cycles.

In this study, each cycle–year trait combination was modeled and predicted independently (e.g. free threshing Year 1 and free threshing Year 2 were not predicted together). Current work is focused on incorporating all data into one model, regardless of year of data observation, accounting for the longitudinal and multi‐year unbalanced nature of the program. Ideally, this will result in selecting stable genotypes that have consistent performance across multiple years. Another unknown that could be answered is if the rate of genetic gain can be maintained across many cycles of selection.

Although the data presented support continued use of GS, we do not have a completely randomized experiment to measure realized genetic gain per cycle. As more cycles of selection are completed, we also anticipate conducting a truly randomized generational experiment with appropriate replications to evaluate genetic gain between cycles. This will allow all cycles to be compared in the same environment and at the same growth stages, which cannot currently be completed within the normal flow of the the breeding program.

## 5 CONCLUSIONS

Development of new perennial crops for sustainable agriculture is a visionary yet daunting task (Crews et al., 2018; Glover et al., 2010). New technologies and genomic tools can be used to speed the process. The rapid cycling enabled by GS, 1 yr per cycle vs. two or more years, will accelerate progress in developing IWG as a perennial grain crop. Our results show that GS is effective at improving traits in IWG and, for some traits, like free threshing, can more than double progress from phenotypic selection. As key domestication traits—free threshing, shattering, and seed mass—are improved, more efforts can be devoted to selection efforts for spike yield and other agronomic traits. This should result in higher genetic improvement as selection on many traits slows progress relative to selecting on one or a few traits (DeHaan et al., 2018). Additionally, the use of validation populations over many years allows for selection on perennial traits without lengthening the breeding cycle and reducing genetic gain per year. Sustained breeding efforts should be able to develop perennial crops that can produce food and fiber while simultaneously providing beneficial ecosystem services.

## CONFLICT OF INTEREST

The authors declare that they have no conflict of interest. The founding sponsors had no role in experimental design, data collection, analysis, interpretation of results, or the decision to publish results.

## AUTHOR CONTRIBUTION

JC, LD, and JP provided conceptualization and methodology. LD conducted investigations and provided plant resources. JC conducted formal analysis and prepared the original draft with all authors reviewing, editing, and approving the final manuscript.

## Supporting information


**Supplemental Table S1**. Number of individuals and single nucleotide polymorphisms (SNPs) before and after filtering for each Cycle of selection.
**Supplemental Table S2**. Validation populations for TLI‐Cycle 7 and TLI‐Cycle 8. The validation population was grown at two different sites (KEB or KOH), with traits being collected in multiple years.

## References

[tpg220080-bib-0001] Bajgain, P. , Zhang, X. , & Anderson, J. A. (2019). Genome‐wide association study of yield component traits in intermediate wheatgrass and implications in genomic selection and breeding. G3: Genes, Genomes, Genetics, 9, 2429–2439. 10.1534/g3.119.400073 31147390 PMC6686922

[tpg220080-bib-0002] Bajgain, P. , Zhang, X. , & Anderson, J. A. (2020). Dominance and G×E interaction effects improve genomic prediction and genetic gain in intermediate wheatgrass (Thinopyrum intermedium). Plant Genome, 13, e20012. 10.1002/tpg2.20012 33016625 PMC12806932

[tpg220080-bib-0003] Browning, B. L. , & Browning, S. R. (2016). Genotype imputation with millions of reference samples. American Journal of Human Genetics, 98, 116–126. 10.1016/j.ajhg.2015.11.020 26748515 PMC4716681

[tpg220080-bib-0004] Calus, M. P. L. (2010). Genomic breeding value prediction: Methods and procedures. Animal, 4, 8. 10.1017/S1751731109991352 22443868

[tpg220080-bib-0005] Cox, T. S. , Van Tassel, D. L. , Cox, C. M. , & Dehaan, L. R. (2010). Progress in breeding perennial grains. Crop Pasture Science, 61, 513–521. 10.1071/CP0920

[tpg220080-bib-0006] Crain, J. , Bajgain, P. , Anderson, J. , Zhang, X. , DeHaan, L. , & Poland, J. (2020a). Enhancing crop domestication through genomic selection, a case study of intermediate wheatgrass. Frontiers in Plant Science, 11, 1–15. 10.3389/fpls.2020.00319 32265968 PMC7105684

[tpg220080-bib-0007] Crain, J. L. , Haghighattalab, A. , Dehaan, L. , & Poland, J. A. (2021). Genomic selection can increase the rate of genetic gain in intermediate wheatgrass (*Thinopyrum intermedium*) breeding. Plant Genome (in press). 10.1002/tpg2.20089 PMC12807048 33900690

[tpg220080-bib-0008] Crews, T. E. , Carton, W. , & Olsson, L. (2018). Is the future of agriculture perennial? Imperatives and opportunities to reinvent agriculture by shifting from annual monocultures to perennial polycultures. Global Sustainability, 1, E11. 10.1017/sus.2018.11

[tpg220080-bib-0009] Culman, S. W. , Snapp, S. S. , Ollenburger, M. , Basso, B. , & DeHaan, L. R. (2013). Soil and water quality rapidly responds to the perennial grain Kernza wheatgrass. Agronomy Journal, 105, 735–744. 10.2134/agronj2012.0273

[tpg220080-bib-0010] DeHaan, L. , Christians, M. , Crain, J. , & Poland, J. (2018). Development and evolution of an intermediate wheatgrass domestication program. Sustainability, 10, 1499. 10.3390/su10051499

[tpg220080-bib-0011] DeHaan, L. R. , & Ismail, B. P. (2017). Perennial cereals provide ecosystem benefits. Cereal Foods World, 62, 278–281. 10.1094/CFW-62-6-0278

[tpg220080-bib-0012] DeHaan, L. R. , Wang, S. , Larson, S. R. , Cattani, D. J. , Zhang, X. , Viinanen, T. (2014). Current efforts to develop perennial wheat and domesticate *Thinopyrum intermedium* as a perennial grain. In: Batello, C. , Wade L. , Cox S. , Pogna N. , Bozzini A. , & Choptiany J. (Eds.), Perennial crops for food security: Proceedings of the FAO expert workshop (pp. 72–89). Rome, Italy: Food and Agriculture Organization of the United Nations.

[tpg220080-bib-0013] de Mendibury, F. (2020). agricolae: Statistical procedures for agricultural research. Retrieved from https://cran.r-project.org/package=agricolae

[tpg220080-bib-0014] Endelman, J. B. (2011). Ridge regression and other kernels for genomic selection with R package rrBLUP. Plant Genome, 4, 250–255. 10.3835/plantgenome2011.08.0024

[tpg220080-bib-0015] Endelman, J. B. , & Jannink, J. L. (2012). Shrinkage estimation of the realized relationship matrix. G3: Genes, Genomes, Genetics, 2, 1405–1413. 10.1534/g3.112.004259 23173092 PMC3484671

[tpg220080-bib-0016] Falconer, D. S. , & Mackay, T. F. (1996). Introduction to quantitative genetics. Essex, UK: Addison Wesley Longman.

[tpg220080-bib-0017] Fletcher, T. D. (2010). Psychometric: Applied psychometric theory . Retrieved from https://cran.r-project.org/package=psychometric

[tpg220080-bib-0018] Gilmour, A. R. , Gogel, B. J. , Cullis, B. R. , Welham, S. J. , & Thompson, R. (2015). ASReml user guide release 4.1, functional specification. Hemel, Hempstead, UK: VSN International Ltd.

[tpg220080-bib-0019] Glaubitz, J. C. J. , Casstevens, T. M. T. , Lu, F. , Harriman, J. , Elshire, R. R. J. , Sun, Q. , & Buckler, E. S. (2014). TASSEL‐GBS: A high capacity genotyping by sequencing analysis pipeline. PLoS ONE, 9, e90346. 10.1371/journal.pone.0090346 24587335 PMC3938676

[tpg220080-bib-0020] Glover, J. D. , Reganold, J. P. , Bell, L. W. , Borevitz, J. , Brummer, E. C. , Buckler, E. S. , Cox, C. M. , Cox, T. S. , Crews, T. E. , Culman, S. W. , DeHaan, L. R. , Eriksson, D. , Gill, B. S. , Holland, J. , Hu, F. , Hulke, B. S. , Ibrahim, A. M. H. , Jackson, W., Jones, S. S. , … Xu, Y. (2010). Increased food and ecosystem security via perennial grains. Science, 328, 1638–1639. 10.1126/science.1188761 20576874

[tpg220080-bib-0021] Godfray, H. C. J. , Beddington, J. R. , Crute, I. R. , Haddad, L. , Lawrence, D. , Muir, J. F. , … Toulmin, C. (2012). Food security: The challenge of feeding 9 billion people. Science, 327, 812–818. 10.1126/science.1185383. 20110467

[tpg220080-bib-0022] Heffner, E. L. , Lorenz, A. J. , Jannink, J. L. , & Sorrells, M. E. (2010). Plant breeding with genomic selection: Gain per unit time and cost. Crop Science, 50, 1681–1690. 10.2135/cropsci2009.11.0662

[tpg220080-bib-0023] Isik, F. , Holland, J. , & Maltecca, C. (2017). Genetic data analysis for plant and animal breeding. Cham, Switzerland: Springer International.

[tpg220080-bib-0024] Jordan, G. J. , Potts, B. M. , & Wiltshire, R. J. E. (1999). Strong, independent, quantitative genetic control of the timing of vegetative phase change and first flowering in *Eucalyptus globulus* ssp. *globulus* (Tasmanian Blue Gum). Heredity, 83, 179–187. 10.1046/j.1365-2540.1999.00570.x 10469206

[tpg220080-bib-0025] Jungers, J. M. , DeHaan, L. H. , Mulla, D. J. , Sheaffer, C. C. , & Wyse, D. L. (2019). Reduced nitrate leaching in a perennial grain crop compared to maize in the Upper Midwest, USA. Agriculture Ecosystems and Environment, 272, 63–73. 10.1016/j.agee.2018.11.007

[tpg220080-bib-0026] Lynch, M. , & Walsh, B. (1998). Genetics and analysis of quantitative traits. Sunderland, MA: Sinauer Associates, Inc.

[tpg220080-bib-0027] Marshall, A. M. , & Wilkins, R. W. (2003). Improved seed yield in perennial ryegrass (Lolium perenne L.) from two generations of phenotypic selection. Euphytica, 133, 233–241. 10.1023/A:1025593808010

[tpg220080-bib-0028] Meuwissen, T. H. E. , Hayes, B. J. , & Goddard, M. E. (2001). Prediction of total genetic value using genome‐wide dense marker maps. Genetics, 157, 1819–1829. 11290733 10.1093/genetics/157.4.1819 PMC1461589

[tpg220080-bib-0029] Mrode, R. A. (2005). Linear models for the prediction of animal breeding values (3rd ed.). Boston, MA: Centre for Agriculture and Bioscience International.

[tpg220080-bib-0030] Poland, J. A. , Brown, P. J. , Sorrells, M. E. , & Jannink, J. L. (2012). Development of high‐density genetic maps for barley and wheat using a novel two‐enzyme genotyping‐by‐sequencing approach. PLoS ONE, 7, e32253. 10.1371/journal.pone.0032253 22389690 PMC3289635

[tpg220080-bib-0031] Sprunger, C. D. , Culman, S. W. , Robertson, G. P. , & Snapp, S. S. (2018). Perennial grain on a Midwest Alfisol shows no sign of early soil carbon gain. Renewable Agriculture and Food Systems, 33, 360–372. 10.1017/S1742170517000138

[tpg220080-bib-0032] Tilman, D. , Balzer, C. , Hill, J. , & Befort, B. L. (2011). Global food demand and the sustainable intensification of agriculture. Proceedings of the National Academy of Sciences of the United States of America, 108, 20260–20264. 10.1073/pnas.1116437108. 22106295 PMC3250154

[tpg220080-bib-0033] Wagoner, P. (1990). Perennial grain development: Past efforts and potential for the future. Critical Reviews in Plant Sciences, 9, 381–408. 10.1080/07352689009382298

[tpg220080-bib-0034] Wong, C. K. , & Bernardo, R. (2008). Genomewide selection in oil palm: Increasing selection gain per unit time and cost with small populations. Theoretical and Applied Genetics, 116, 815–824. 10.1007/s00122-008-0715-5 18219476

[tpg220080-bib-0035] Zhang, X. , Sallam, A. , Gao, L. , Kantarski, T. , Poland, J. , DeHaan, L. R. , Donald, L. , & Anderson, J. A. (2016). Establishment and optimization of genomic selection to accelerate the domestication and improvement of intermediate wheatgrass. Plant Genome, 9, 1–18. 10.3835/plantgenome2015.07.0059 27898759

