# Supplementary material for: Genomic prediction enables rapid selection of high‐performing genets in an intermediate wheatgrass breeding program
Source: Plant Genome. 2021 Mar 3;14(2):e20080. doi: 10.1002/tpg2.20080 (PMC12899483; doi:10.1002/tpg2.20080)
Supplement: Supplementary file 1 [file TPG2-14-e20080-s001.docx]

Supplemental Material

**Supplementary Table S1**. Number of individuals and single nucleotide polymorphisms (SNPs) before and after filtering for each Cycle of selection.

| **Cycle** | **Total SNPs** | Training Population | | | Prediction Population | | |
| --- | --- | --- | --- | --- | --- | --- | --- |
|  |  | SNPs | Individuals | | SNPs | Individuals | |
|  |  | After Filtering | Before Filtering | After Filtering | After Filtering | Before Filtering | After Filtering |
| TLI-Cycle 7 | 132098 | 18355 | 3040 | 2951 | 22697 | 7210 | 6998 |
| TLI-Cycle 8 | 118166 | 26021 | 7743 | 7680 | 18960 | 8360 | 6665 |
| TLI-Cycle 9 | 121370 | 21957 | 13194 | 11853 | 19401 | 10617 | 10084 |

**Supplementary Table S2.** Validation populations for TLI-Cycle 7 and TLI-Cycle 8. The validation population was grown at two different sites (KEB or KOH), with traits being collected in multiple years. Traits, number of observations (n), mean, standard deviation (SD), and range of observed are listed for phenotypic observations. Priority traits are in bold.

| **Cycle** | **Site** | **Year** | **Trait** | **n** | **Mean (SD)** | **Range** |
| --- | --- | --- | --- | --- | --- | --- |
| TLI-Cycle 7 | KEB | 2019 | Brittle Rachis | 686 | 0.44 (0.5) | 0 — 1 |
| TLI-Cycle 7 | KOH | 2019 | Brittle Rachis | 694 | 0.18 (0.38) | 0 — 1 |
| TLI-Cycle 8 | KEB | 2019 | Brittle Rachis | 9 | 1 (0) | 1 — 1 |
| TLI-Cycle 8 | KOH | 2019 | Brittle Rachis | 6 | 1 (0) | 1 — 1 |
| **TLI-Cycle 7** | **KEB** | **2018** | **Free Threshing** | **467** | **71.95 (23.37)** | **0** — **100** |
| **TLI-Cycle 7** | **KOH** | **2018** | **Free Threshing** | **384** | **76.41 (21.69)** | **0** — **100** |
| **TLI-Cycle 7** | **KEB** | **2019** | **Free Threshing** | **684** | **59.25 (23.3)** | **0 — 100** |
| **TLI-Cycle 7** | **KOH** | **2019** | **Free Threshing** | **692** | **59.14 (24.52)** | **0 — 100** |
| **TLI-Cycle 8** | **KEB** | **2019** | **Free Threshing** | **485** | **66.3 (18.94)** | **4 — 100** |
| **TLI-Cycle 8** | **KOH** | **2019** | **Free Threshing** | **393** | **70.48 (16.68)** | **7 — 100** |
| TLI-Cycle 7 | KEB | 2019 | Lodging | 678 | 5.9 (2.35) | 0 — 10 |
| TLI-Cycle 7 | KOH | 2019 | Lodging | 692 | 6.07 (2.05) | 0 — 10 |
| TLI-Cycle 7 | KEB | 2018 | Maturity | 572 | 56.78 (5.06) | 30 — 69 |
| TLI-Cycle 7 | KOH | 2018 | Maturity | 586 | 57.28 (3.38) | 45 — 69 |
| TLI-Cycle 7 | KEB | 2019 | Maturity | 687 | 68.96 (2.92) | 55 — 77 |
| TLI-Cycle 7 | KOH | 2019 | Maturity | 691 | 60.82 (3.15) | 51 — 71 |
| TLI-Cycle 7 | KEB | 2018 | Number of Florets per Spike | 559 | 190.46 (43.43) | 56 — 325 |
| TLI-Cycle 7 | KOH | 2018 | Number of Florets per Spike | 582 | 201.68 (45.42) | 84 — 378 |
| TLI-Cycle 7 | KEB | 2019 | Number of Florets per Spike | 686 | 168.28 (46.31) | 54 — 336 |
| TLI-Cycle 7 | KOH | 2019 | Number of Florets per Spike | 693 | 157.21 (42.76) | 48 — 312 |
| TLI-Cycle 8 | KEB | 2019 | Number of Florets per Spike | 486 | 195.84 (42.06) | 55 — 308 |
| TLI-Cycle 8 | KOH | 2019 | Number of Florets per Spike | 393 | 170.63 (38.36) | 64 — 336 |
| TLI-Cycle 7 | KEB | 2018 | Number of Florets per Spikelet | 559 | 8.79 (1.51) | 5 — 15 |
| TLI-Cycle 7 | KOH | 2018 | Number of Florets per Spikelet | 582 | 8.87 (1.53) | 5 — 15 |
| TLI-Cycle 7 | KEB | 2019 | Number of Florets per Spikelet | 686 | 7.13 (1.63) | 3 — 12 |
| TLI-Cycle 7 | KOH | 2019 | Number of Florets per Spikelet | 693 | 6.71 (1.48) | 3 — 12 |
| TLI-Cycle 8 | KEB | 2019 | Number of Florets per Spikelet | 486 | 8.81 (1.53) | 5 — 14 |
| TLI-Cycle 8 | KOH | 2019 | Number of Florets per Spikelet | 393 | 7.99 (1.29) | 4 — 12 |
| TLI-Cycle 7 | KEB | 2019 | Peduncle Width | 679 | 9.99 (1.33) | 6 — 14.5 |
| TLI-Cycle 7 | KOH | 2019 | Peduncle Width | 682 | 10.06 (1.31) | 6 — 14 |
| TLI-Cycle 7 | KEB | 2018 | Percent Seed Set | 555 | 0.12 (0.11) | 0 — 0.61 |
| TLI-Cycle 7 | KOH | 2018 | Percent Seed Set | 580 | 0.03 (0.04) | 0 — 0.27 |
| TLI-Cycle 7 | KEB | 2019 | Percent Seed Set | 682 | 0.34 (0.15) | 0.01 — 0.96 |
| TLI-Cycle 7 | KOH | 2019 | Percent Seed Set | 691 | 0.35 (0.14) | 0.01 — 0.85 |
| TLI-Cycle 8 | KEB | 2019 | Percent Seed Set | 479 | 0.24 (0.12) | 0.01 — 0.63 |
| TLI-Cycle 8 | KOH | 2019 | Percent Seed Set | 391 | 0.22 (0.11) | 0.01 — 0.64 |
| TLI-Cycle 7 | KEB | 2018 | Plant Height | 566 | 86.67 (13.76) | 35 — 125 |
| TLI-Cycle 7 | KOH | 2018 | Plant Height | 587 | 84.37 (12.15) | 50 — 115 |
| TLI-Cycle 7 | KEB | 2019 | Plant Height | 687 | 140.39 (15.05) | 80 — 180 |
| TLI-Cycle 7 | KOH | 2019 | Plant Height | 699 | 131.66 (14.5) | 80 — 190 |
| TLI-Cycle 8 | KEB | 2019 | Plant Height | 497 | 99.86 (15.09) | 50 — 140 |
| TLI-Cycle 8 | KOH | 2019 | Plant Height | 470 | 89.21 (14.61) | 40 — 135 |
| TLI-Cycle 7 | KEB | 2018 | Seed Area | 467 | 8.69 (1.05) | 5.81 — 11.76 |
| TLI-Cycle 7 | KOH | 2018 | Seed Area | 384 | 8.2 (0.99) | 5.55 — 10.91 |
| TLI-Cycle 7 | KEB | 2019 | Seed Area | 684 | 7.57 (1.1) | 3.46 — 10.82 |
| TLI-Cycle 7 | KOH | 2019 | Seed Area | 692 | 8.32 (1.13) | 5.47 — 13.09 |
| TLI-Cycle 8 | KEB | 2019 | Seed Area | 482 | 7.58 (1.24) | 4.4 — 11.8 |
| TLI-Cycle 8 | KOH | 2019 | Seed Area | 391 | 7.45 (1.13) | 4.54 — 11.87 |
| TLI-Cycle 7 | KEB | 2018 | Seed Density | 467 | 1.42 (0.21) | 0.81 — 1.99 |
| TLI-Cycle 7 | KOH | 2018 | Seed Density | 381 | 1.33 (0.2) | 0.75 — 1.87 |
| TLI-Cycle 7 | KEB | 2019 | Seed Density | 682 | 1.49 (0.27) | 0.5 — 2.37 |
| TLI-Cycle 7 | KOH | 2019 | Seed Density | 692 | 1.53 (0.23) | 0.71 — 2.41 |
| TLI-Cycle 8 | KEB | 2019 | Seed Density | 482 | 1.54 (0.21) | 0.88 — 2.07 |
| TLI-Cycle 8 | KOH | 2019 | Seed Density | 391 | 1.53 (0.2) | 0.95 — 2.17 |
| TLI-Cycle 7 | KEB | 2018 | Seed Length | 467 | 6.54 (0.55) | 5.04 — 8.1 |
| TLI-Cycle 7 | KOH | 2018 | Seed Length | 384 | 6.23 (0.51) | 4.62 — 7.51 |
| TLI-Cycle 7 | KEB | 2019 | Seed Length | 684 | 6.46 (0.59) | 4.06 — 8.11 |
| TLI-Cycle 7 | KOH | 2019 | Seed Length | 692 | 6.63 (0.61) | 4.83 — 8.78 |
| TLI-Cycle 8 | KEB | 2019 | Seed Length | 482 | 6.53 (0.56) | 4.8 — 8.23 |
| TLI-Cycle 8 | KOH | 2019 | Seed Length | 391 | 6.51 (0.53) | 4.77 — 8.64 |
| **TLI-Cycle 7** | **KEB** | **2018** | **Seed Mass** | **467** | **9.33 (1.75)** | **4.87 — 14.65** |
| **TLI-Cycle 7** | **KOH** | **2018** | **Seed Mass** | **381** | **8.32 (1.54)** | **4 — 12.79** |
| **TLI-Cycle 7** | **KEB** | **2019** | **Seed Mass** | **682** | **9.65 (2.1)** | **2.87 — 16.2** |
| **TLI-Cycle 7** | **KOH** | **2019** | **Seed Mass** | **692** | **10.16 (2.01)** | **4.17 — 17.47** |
| **TLI-Cycle 8** | **KEB** | **2019** | **Seed Mass** | **482** | **10.08 (1.7)** | **4.76 — 16.25** |
| **TLI-Cycle 8** | **KOH** | **2019** | **Seed Mass** | **391** | **9.98 (1.72)** | **4.83 — 15.65** |
| TLI-Cycle 7 | KEB | 2018 | Seed Width | 467 | 1.72 (0.12) | 1.32 — 2.03 |
| TLI-Cycle 7 | KOH | 2018 | Seed Width | 384 | 1.7 (0.11) | 1.34 — 1.96 |
| TLI-Cycle 7 | KEB | 2019 | Seed Width | 684 | 1.51 (0.13) | 1.13 — 1.93 |
| TLI-Cycle 7 | KOH | 2019 | Seed Width | 692 | 1.63 (0.12) | 1.32 — 2.02 |
| TLI-Cycle 8 | KEB | 2019 | Seed Width | 482 | 1.51 (0.16) | 1.12 — 1.93 |
| TLI-Cycle 8 | KOH | 2019 | Seed Width | 391 | 1.49 (0.13) | 1.09 — 1.79 |
| TLI-Cycle 7 | KEB | 2018 | Seeds per Spike | 556 | 23.47 (21.72) | 0 — 98.78 |
| TLI-Cycle 7 | KOH | 2018 | Seeds per Spike | 580 | 5.52 (7.48) | 0 — 46.1 |
| TLI-Cycle 7 | KEB | 2019 | Seeds per Spike | 682 | 54.6 (22.28) | 1.04 — 131.91 |
| TLI-Cycle 7 | KOH | 2019 | Seeds per Spike | 692 | 52.9 (21) | 1.13 — 126.24 |
| TLI-Cycle 8 | KEB | 2019 | Seeds per Spike | 479 | 46.26 (21.1) | 1.04 — 114.88 |
| TLI-Cycle 8 | KOH | 2019 | Seeds per Spike | 391 | 36.3 (18) | 0.75 — 97.25 |
| **TLI-Cycle 7** | **KEB** | **2018** | **Shattering** | **560** | **1.42 (1.03)** | **0 — 4** |
| **TLI-Cycle 7** | **KOH** | **2018** | **Shattering** | **582** | **1.4 (0.79)** | **0 — 3.8** |
| **TLI-Cycle 7** | **KEB** | **2019** | **Shattering** | **685** | **2.66 (1.09)** | **0 — 4** |
| **TLI-Cycle 7** | **KOH** | **2019** | **Shattering** | **694** | **2.39 (1.23)** | **0 — 4** |
| **TLI-Cycle 8** | **KEB** | **2019** | **Shattering** | **486** | **2.41 (0.89)** | **0 — 4** |
| **TLI-Cycle 8** | **KOH** | **2019** | **Shattering** | **393** | **2.15 (0.94)** | **0 — 4** |
| TLI-Cycle 7 | KEB | 2018 | Spike Emergence | 584 | 18.64 (11.33) | 0 — 45 |
| TLI-Cycle 7 | KEB | 2018 | Spike Emergence | 556 | 0.6 (0.34) | 0 — 1.61 |
| TLI-Cycle 7 | KOH | 2018 | Spike Emergence | 598 | 20.89 (11.12) | 0 — 50 |
| TLI-Cycle 7 | KOH | 2018 | Spike Emergence | 576 | 0.63 (0.31) | 0 — 1.6 |
| TLI-Cycle 7 | KEB | 2018 | Spike Length | 560 | 32.41 (4.91) | 0 — 52 |
| TLI-Cycle 7 | KOH | 2018 | Spike Length | 583 | 34.32 (5.08) | 3 — 49 |
| **TLI-Cycle 7** | **KEB** | **2018** | **Spike Yield** | **560** | **0.22 (0.2)** | **0 — 0.92** |
| **TLI-Cycle 7** | **KOH** | **2018** | **Spike Yield** | **582** | **0.05 (0.07)** | **0 — 0.47** |
| **TLI-Cycle 7** | **KEB** | **2019** | **Spike Yield** | **686** | **0.52 (0.23)** | **0 — 1.33** |
| **TLI-Cycle 7** | **KOH** | **2019** | **Spike Yield** | **694** | **0.53 (0.22)** | **0 — 1.21** |
| **TLI-Cycle 8** | **KEB** | **2019** | **Spike Yield** | **483** | **0.47 (0.23)** | **0 — 1.14** |
| **TLI-Cycle 8** | **KOH** | **2019** | **Spike Yield** | **393** | **0.36 (0.18)** | **0 — 0.87** |
| TLI-Cycle 7 | KEB | 2018 | Spikelets per Inflorescence | 559 | 21.64 (3.02) | 8 — 29 |
| TLI-Cycle 7 | KOH | 2018 | Spikelets per Inflorescence | 582 | 22.65 (2.73) | 11 — 32 |
| TLI-Cycle 7 | KEB | 2019 | Spikelets per Inflorescence | 686 | 23.56 (3.43) | 11 — 32 |
| TLI-Cycle 7 | KOH | 2019 | Spikelets per Inflorescence | 694 | 23.32 (3.08) | 13 — 34 |
| TLI-Cycle 8 | KEB | 2019 | Spikelets per Inflorescence | 486 | 22.21 (2.81) | 11 — 31 |
| TLI-Cycle 8 | KOH | 2019 | Spikelets per Inflorescence | 393 | 21.25 (2.64) | 11 — 28 |
| TLI-Cycle 7 | KEB | 2018 | Stem Angle | 583 | 53.95 (20.15) | 0 — 85 |
| TLI-Cycle 7 | KOH | 2018 | Stem Angle | 598 | 48.52 (19.72) | 5 — 90 |
| TLI-Cycle 7 | KEB | 2018 | Stem Strength | 493 | 1414.31 (469.1) | 430 — 3284 |
| TLI-Cycle 7 | KOH | 2018 | Stem Strength | 504 | 1662.44 (540.22) | 549 — 4925 |
| TLI-Cycle 7 | KEB | 2019 | Stem Strength | 686 | 932.19 (473.32) | 22 — 3989 |
| TLI-Cycle 7 | KOH | 2019 | Stem Strength | 697 | 989.4 (423.88) | 123 — 3117 |
